# Background-deflection Brillouin microscopy reveals altered biomechanics of intracellular stress granules by ALS protein FUS

**DOI:** 10.1038/s42003-018-0148-x

**Published:** 2018-09-10

**Authors:** Giuseppe Antonacci, Valeria de Turris, Alessandro Rosa, Giancarlo Ruocco

**Affiliations:** 1 Center for Life Nano Science@Sapienza, Istituto Italiano di Tecnologia, Rome, Italy; 2 Department of Biology and Biotechnology Charles Darwin, University of Rome”Sapienza”, Rome, Italy; 3 Department of Physics, University of Rome ”Sapienza”, Rome, Italy

## Abstract

Altered cellular biomechanics have been implicated as key photogenic triggers in age-related diseases. An aberrant liquid-to-solid phase transition, observed in in vitro reconstituted droplets of FUS protein, has been recently proposed as a possible pathogenic mechanism for amyotrophic lateral sclerosis (ALS). Whether such transition occurs in cell environments is currently unknown as a consequence of the limited measuring capability of the existing techniques, which are invasive or lack of subcellular resolution. Here we developed a non-contact and label-free imaging method, named background-deflection Brillouin microscopy, to investigate the three-dimensional intracellular biomechanics at a sub-micron resolution. Our method exploits diffraction to achieve an unprecedented 10,000-fold enhancement in the spectral contrast of single-stage spectrometers, enabling, to the best of our knowledge, the first direct biomechanical analysis on intracellular stress granules containing ALS mutant FUS protein in fixed cells. Our findings provide fundamental insights on the critical aggregation step underlying the neurodegenerative ALS disease.

## Abstract (web summary)

Giuseppe Antonacci et al. present a method for non-contact and label-free imaging using Brillouin microscopy to examine intracellular biomechanics at the sub-micron level. They use this approach in an analysis of stress granules containing ALS-related FUS protein mutants.

## Introduction

Demand to reveal fundamental micro-mechanical properties is driven by growing evidence that altered cellular processes in aging-associated disease environments are caused by a change in the regulating biomechanics. Persuasive evidence suggests that the altered cellular motility in cancer metastasis is directly associated with a change in the cytoskeleton stiffness, leading a modified contractility that promotes cellular migration^1^. In cardiovascular diseases, wall stiffness determines rupture of the atherosclerotic plaques caused by progressive extracellular lipid accumulation and calcification^2^. Moreover, a decreased cerebral viscoelasticity has been recently observed in multiple sclerosis^3^, suggesting that altered biomechanics of neurons or glial cells contribute to neuroinflammation.

Recent analysis has further pointed to aberrant phase transition as a pathogenic mechanism underlying the neurodegenerative disease amyotrophic lateral sclerosis (ALS)^4^. Mutations in prion-like proteins with a low-sequence complexity domain, such as FUS, have been recently associated with familial ALS^5^. These proteins are involved in the formation of membrane-less compartments by a liquid–liquid phase separation mechanism in the cell. An example of such compartments is represented by stress granules, cytoplasmic structures containing RNA and RNA-binding proteins. Stress granule formation is induced in eukaryotic cells exposed to environmental insults, such as oxidative stress, as a pro-survival mechanism enabling the temporary storage of housekeeping mRNAs. At the same time, the synthesis of stress-protective factors, such as heat-shock proteins and chaperones, is allowed^6^. Upon recovery from stress, stress granules are disassembled and general translation re-initiated. ALS-linked mutations in FUS cause a shift in its localization from the nucleus to the cytoplasm and its recruitment into stress granules. Interestingly, a liquid-to-solid phase transition occurs in vitro in reconstituted liquid droplets containing mutated FUS^7,8^. It has been proposed that such transition represents an aberrant trigger to the formation of insoluble pathological aggregates found in ALS patients^7,9,10^. Whether such transition occurs also in the cell environment is currently unknown.

Advances in cellular biomechanics and phase transitions have so far been limited by the available measuring methods^11^. Conventional atomic force microscopy (AFM)^12^, optical tweezers^13^, and micropipette aspiration^14^ rely on the application of mechanical forces to the cells, which makes these methods invasive and limited to surface topologies. Other non-contact techniques, such as those based on ultrasounds^15^, magnetic resonance imaging (MRI)^16,17^, and particle tracking^18^, suffer from a poor spatial resolution or require sample labeling. In turn, confocal Brillouin microscopy has been recently proposed^19–21^ to enable a three-dimensional all-optical and label-free investigation of the biomechanical properties of whole cells^22–24^ and tissues^20,25^. In Brillouin microscopy, local spontaneous acoustic waves existing at thermal equilibrium in the sample are probed by a narrow-bandwidth laser source using a scanning confocal microscope, and the Brillouin spectrum of the inelastically scattered light is analyzed by a high-resolution (<1 GHz) virtually imaged phased array (VIPA) spectrometer^26,27^ to measure the real and imaginary part of the high-frequency longitudinal elastic modulus^28^. The contactless mechanism together with the enabled subcellular resolution^29^ have promoted Brillouin microscopy as a unique tool to reveal the missing biomechanical information in the volume of biosystems, promising advances in early and label-free diagnosis of diseases, such as keratoconus^30,31^, atherosclerosis^25^, cancer^32^, Alzheimer’s disease^33^, and bacterial meningitis^34^.

The main challenge in Brillouin microscopy is localizing the Brillouin peaks when strong elastic background light is delivered to the spectrometer. Besides the elastic Rayleigh scattering arising in turbid biological samples, specular Fresnel reflections are dominant when the optical sectioning is performed near the water-glass interface of a sample coverslip. Given the limited spectral contrast of commonly used VIPA spectrometers, an excess amount of elastic background light results in the formation of crosstalk signals along the dispersion axis that overwhelm the weakly scattered Brillouin signal^35^. Existing methods to increase the contrast involve the use of crossed multistage VIPA etalons^35^ or multipass Fabry-Perot interferometers^36,37^, but the enhancement comes at the cost of an increased system complexity with ensuing reduced throughput efficiency. Field apodization is a promising method^22,38^, yet further development is required to avoid optical losses. Other techniques, such as destructive interference^39^, cell absorption^40^, etalon filtering^41^, and dark-field illumination^42^, have been successfully demonstrated in the attempt to suppress the elastic background light, but these leave the contrast of the spectrometer basically unvaried.

Here we present a method that overcomes these limitations, providing an increase in the contrast of a single-stage and single-pass VIPA spectrometer by an unprecedented 10,000-fold without involving additional optical or dispersive elements. Combining the high-contrast spectrometer with a scanning confocal imaging system, we demonstrate the enhanced detection capability of the background-deflection Brillouin (BDB) microscope acquiring Brillouin spectra of turbid media and under critical experimental conditions where the Brillouin signal is typically overwhelmed. Imaging whole cells at a sub-micron resolution, our BDB microscope enabled intracellular investigation of the biomechanical properties of stress granules, suggesting that an increase in both stiffness and viscosity occurs upon inclusion of ALS mutated FUS protein, in line with the liquid-to-solid phase transition previously observed in reconstituted liquid FUS compartments in vitro^7^.

## Results

### Spectral contrast enhancement by background deflection

**Fig. 1:**
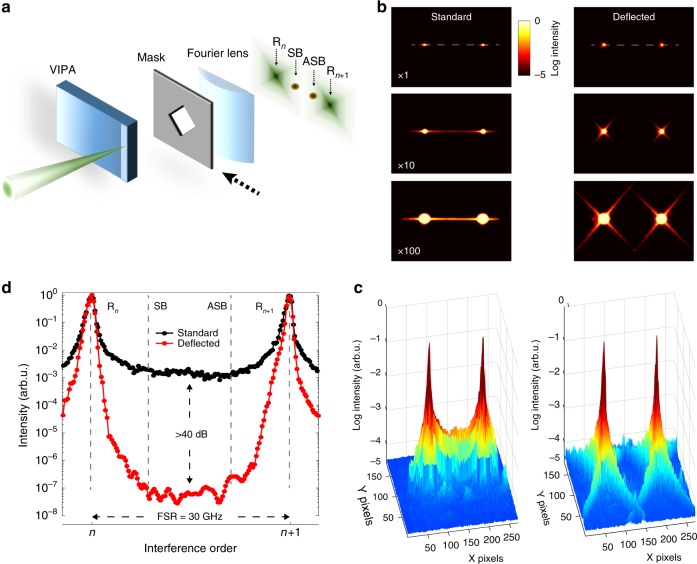
Spectral contrast enhancement by background deflection. **a** Schematic of the spectrometer. The light to be analyzed is focused and coupled to the VIPA through an anti- reflection coated window. A mask with a rhomboidal aperture is placed before the Fourier lens to convolve the resulting diffraction pattern with the intensity transfer function of the VIPA etalon. As a result, the Stokes (SB) and Anti-Stokes (ASB) Brillouin spectral features gain high visibility despite the presence of strong elastic Rayleigh (R) peaks. **b** Interference patterns generated in response to a monochromatic light beam with (right) and without (left) the diffraction mask for different incident optical powers. In the standard configuration, a strong crosstalk line arises along the horizontal dispersion axis (dashed line). On the other hand, the elastic background is highly deflected by the mask, as further illustrated by the 3D plots (**c**). **d** Spectral intensity profiles along the dispersion axis (where the SB and ASB peaks are expected) for two consecutive interference orders. Whilst the standard single-stage VIPA spectrometer (black line) reaches a maximum contrast of ∼10^3^, our spectrometer (red line) gives ∼10^7^, which represents a 10,000-fold increase with respect to the standard case

To address the limited contrast of commonly used VIPA etalons, we aimed to exploit diffraction by a properly shaped mask to strongly deflect the elastic background light from the dispersion axis with low excess losses. Unlike the standard setup, our spectrometer integrates a mask with a rhomboidal-shaped aperture at the cylindrical Fourier lens (Fig. 1a). The advantage of the mask comes from the lens convolution of the well-known intensity transfer function of the VIPA etalon with the diffraction pattern generated by the aperture (Supplementary Fig. 1). By choosing an appropriate mask orientation, the high-energy diffraction tails spread at an angle with respect to the dispersion axis of the VIPA etalon, where the Brillouin peaks are located. Figure 1b compares the interference patterns generated by the standard and our background-deflected spectrometer. At low optical power, the two subsequent interfering orders are clearly separated by one free spectral range along the dispersion axis. However, a pronounced horizontal crosstalk signal appears between the two elastic peaks as the laser intensity increases. On the other hand, the elastic background light is highly deflected from the dispersion axis by the diffraction mask, as further illustrated by the 3D plots (Fig. 1c). The deflected background light results in an unprecedented increase in the contrast given by the spectrometer (Fig. 1d). While the standard single-stage VIPA spectrometer reaches up to ∼10^3^ peak-to-background ratio, our instrument readily gives a contrast of ∼10^7^ without loss in spectral resolution (Supplementary Fig. 2) and ∼ 2.5 dB estimated excess losses.

### Improved detection capability of BDB microscopy

**Fig. 2:**
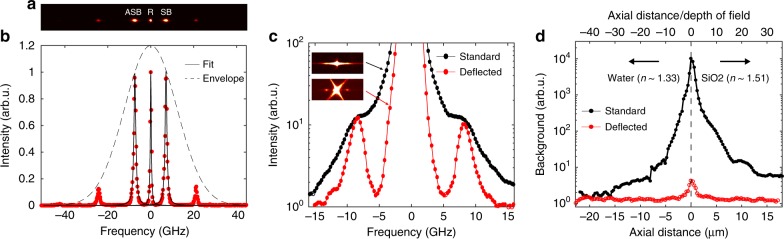
Validation of contrast enhancement. Representative 2D (**a**) and 1D (**b**) Brillouin spectrum of water. **c** Brillouin spectrum of an intralipid solution at concentration of 10%. In a standard VIPA spectrometer (black line), the Brillouin peaks are overwhelmed by the light scattered elastically. The spectrum, however, becomes visible (red line) when the background is deflected. **d** Background of the standard (black line) and deflected (red line) spectrometers averaged on a spectral range of 5–25 GHz as a function of the distance from a water-glass interface (dashed line)

The enhanced contrast and detection capability of our BDB microscope was first validated in critical experimental conditions in which strong elastic background signals arise from either highly scattering media or unavoidable specular Fresnel reflections. Figure 2a, b shows a representative Brillouin spectrum of distilled water acquired by the BDB microscope, which integrates the high-contrast VIPA spectrometer in a custom-built confocal microscope of ∼0.3 × 0.3 × 1.1 μm^3^ spatial resolution (Methods and Supplementary Fig. 3). The shift of the stokes and anti-stokes Brillouin peaks was measured to be *ν*_B_ = 7.41 ± 0.01 GHz, in agreement with previous reports^20^. The visibility of the Brillouin peaks rapidly decreases as the turbidity of the medium increases. At 10% concentration of intralipid solution, detection with the standard configuration is impeded by the overwhelming elastic crosstalk signal (Fig. 2c). The spectrum, however, becomes clearly visible when the elastic background light is deflected from the dispersion axis, demonstrating the instrumental ability to perform measurements even in the presence of high turbidity. We further measured the mean instrumental background intensity along the dispersion axis through a water-glass interface (Fig. 2d), simulating a typical experimental condition in which the optical sectioning is performed nearby a sample coverslip. In the standard case, the background signal sharply increases by four orders of magnitude within a 5 μm range from the glass surface, corresponding to the region where cells are optically probed. On the other hand, our instrument provides a constantly low background signal along the optical axis, with minimal increase at <2 μm from the interface.

### BDB microscopy unveils altered stress granule biomechanics in whole cells

**Fig. 3:**
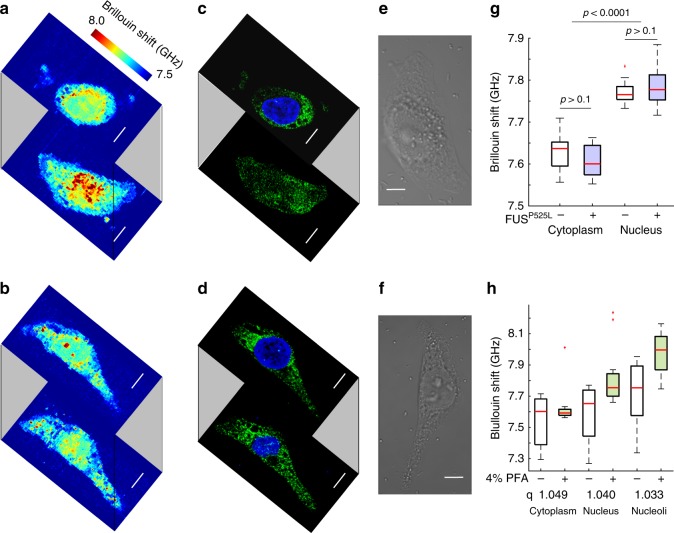
Biomechanical imaging of HeLa cells. The enhanced contrast enabled the acquisition of Brillouin images of single cells at different depths (∆z = 1 μm) in the case of uninduced (**a**) and doxycycline-induced (**b**) cells. Fluorescent images (**c**–**d**) showing the PABP (green) and DNA (blue; labeled by DAPI) merged staining. **e**–**f** Associated DIC images. Scale bar, 10 μm. **g** Box-and-whisker plot of the Brillouin frequency shift for different cellular compartments with (+) and without (−) FUSP525L expression. In both cases the expression of mutant FUS did not significantly altered the cytoplasm (Student’s *t*-test *p* = 0.22, *N* = 25 cells) and the nucleus (*p* = 0.37) properties (**h**) Box-and-whisker plot of the Brillouin frequency shift of living (−) and fixed (+) HeLa cells (*N* = 29, 3 experiments). Despite an overall increase in Brillouin frequency, different subcellular compartments are found to be similarly altered in living and fixed cells as indicated by the frequency ratio q

Our BDB microscope enabled to study the biomechanical properties of intracellular stress granules and their changes in response to mutant FUS recruitment in individual fixed HeLa cells. To this aim, we used a modified HeLa line (Supplementary Note 1 and Supplementary Fig. 4) in which the expression of the mutant FUS protein, tagged with the red fluorescent protein (tagRFP), can be induced by doxycycline (RFP-FUSP525L). Cells were cultured in the presence or absence of doxycycline and in control conditions or upon oxidative stress induction by sodium arsenite (ARS). Cells were imaged with the Brillouin microscope and then with the fluorescence microscope to reveal the location of the mutant FUS (in the red channel), PABP (a stress granules marker stained by immunofluorescence; green channel) and the nucleus (DAPI staining; blue channel). We first analyzed cells in control conditions with or without RFP-FUSP525L expression. Brillouin images of cells cultured in the absence or presence of doxycycline (Fig. 3a, b, respectively) were compared with the corresponding fluorescence (Fig. 3c, d and Supplementary Fig. 5) and differential interference contrast (DIC) images (Fig. 3e, f) to measure the mean Brillouin shift of individual intracellular compartments. In the absence of stress, the Brillouin shifts for both the nucleus and the cytoplasm did not show significant variations (*p* = 0.37 and 0.22, respectively; *N* = 25 cells) upon mutant FUS expression (Fig. 3g).

To assess the effect of 4% PFA fixation on cellular biomechanics, living HeLa cells (*N* = 29, see Methods) were first imaged using the BDB microscope. Another set of measurements was performed after fixation on the same cells and under the original experimental conditions (Fig. 3h). Despite an overall increase in the Brillouin shift (normalized to the cellular volume variations), the frequency ratio *q* = *ν*_fixed_/*ν*_living_ of fixed and living cells was found to be *q* = 1.049 for the cytoplasm, *q* = 1.040 for the nucleus and *q* = 1.033 for the nucleoli. These findings suggest that the entire cells could be evenly altered by the PFA fixation process.

**Fig. 4:**
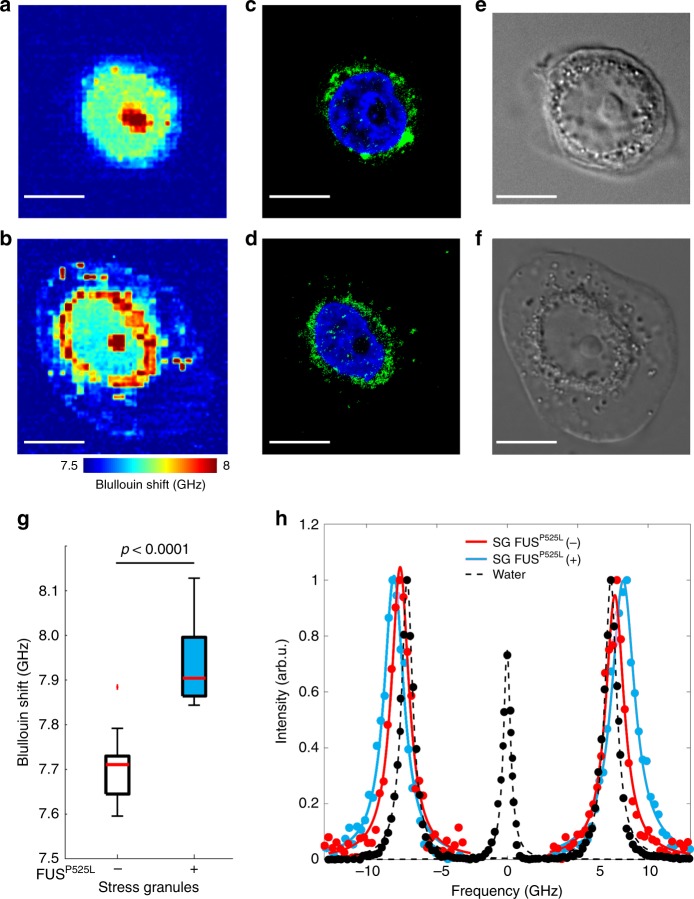
Alteration of stress granule biomechanics. Representative Brillouin z-stack image of stressed HeLa cells without (**a**, Supplementary Video 1 for 3D reconstruction) and with (**b**, Supplementary Video 2) mutant FUS expression. Corresponding fluorescent (**c**–**d**; green: PABP; blue: DAPI) and DIC (**e**–**f**) images. Scale bar, 10 μm. While the nucleoli appear stiffer than the surrounding nucleoplasm in both cases, stress granules manifest a significantly higher (*p* = 2.5 × 10^−8^, *N* = 41 cells on three different experiments) Brillouin shift in response to mutant FUS expression, as illustrated in the box-and-whisker plot (**g**). **h** Representative Brillouin spectra of water (black) and stress granules with (red) and without (green) mutant FUS expression. Besides a higher frequency shift, the Brillouin peaks associated with mutant FUS expression manifests a larger linewidth (∆*ν*_B_ = 1.13 ± 0.02 GHz) than that without (∆*ν*_B_ = 0.86 ± 0.01 GHz), indicating a potential increase in the stress granule viscosity

The analysis on mutant FUS expression was repeated with cells exposed to ARS. Brillouin images were acquired across several z-sections for both untreated (Fig. 4a and Supplementary Video 1) and doxycycline-treated (Fig. 4b and Supplementary Video 2) cells to measure the stiffness of stress granules compartments, which were identified through the associated fluorescent signal (Fig. 4c, d and Supplementary Fig. 6) and DIC (Fig. 4e, f) images. Interestingly, in these conditions we could observe a significant variation (*p* = 2.5 × 10^−8^) in the Brillouin shift specifically associated to the subcellular regions in which stress granules had formed (Fig. 4g and Supplementary Fig. 7). Moreover, the Brillouin spectrum (Fig. 4h) of mutant stress granules manifested broader peaks compared to normal stress granules, suggesting a potential increase in their viscosity. Notably, in the nucleus and in the cytoplasm, we did not observe a significant variation in the Brillouin shift (*p* = 0.32 and 0.24, respectively). Collectively, these results indicate that the biomechanical properties of stress granules were remarkably altered by the inclusion of mutant FUS proteins, resulting in more stiff and viscous (presumably more resistant) aggregates.

## Discussion

A central issue for the cell is related to the internal organization of subcellular compartments that are devoid of membranes. In such structures, variously referred to as granules, bodies or speckles, or collectively as proteinaceous membrane-less organelles, crucial biochemical reactions occur, which must be kept physically separated from the rest of the cytoplasm or nucleoplasm^43^. These proteinaceous membrane-less organelles are established as condensed liquid droplets, formed and maintained through a liquid–liquid phase separation process. The molecular basis of liquid droplets formation relies on low-sequence complexity domain containing prion-like proteins, for which FUS represents a prototypical example. Notably, the usage of these proteins, which invariably contain intrinsically disordered domains, holds downsides for the cell. For instance, mutations in FUS and other on low-sequence complexity domain proteins (and/or their increased concentration) have been proposed to accelerate their aggregation in neurodegenerative diseases, possibly by enhanced stress granule formation^10^ and through a liquid-to-solid phase transition^7^. A better comprehension of the crucial switch between physiological clustering and pathological aggregation is necessary to understand the molecular basis of neurodegeneration. So far, liquid-to-solid transitions have been only observed in liquid FUS compartments reconstituted in vitro and whether similar aberrant phase transitions occur in cells is still unclear^7,8^.

We provide the first direct investigation of the biomechanical properties of intracellular stress granules. Our spectral data show a higher frequency shift *ν*_B_ and a broader linewidth ∆*ν*_B_ of the Brillouin peaks in response to recruitment of the mutant FUS protein, indicating a potential increase in both stiffness and viscosity of the stress granules. While the real part of the longitudinal modulus M′ associated with the Brillouin frequency shift is a measure of stiffness and does not provide information about the physical state of matter, the imaginary part M′′ obtained from the Brillouin linewidth is well-known to be associated with the viscoelastic properties of the material analyzed and thus provides fundamental insights on the condensation state^44^. As such, our findings may suggest the onset of a liquid-to-solid phase transition in stress granules in response to recruitment of the ALS-linked FUS protein, a process that was previously observed in reconstituted liquid FUS compartments in vitro^7^.

This analysis was enabled imaging whole HeLa cells at high spatial and spectral resolution with the newly developed non-contact and label-free BDB microscope. Unlike multistage spectral analysis approaches, our instrument readily achieves a 10,000-fold increase in the spectral contrast without involving additional optical or dispersive elements, providing a robust and more efficient configuration to perform rapid three-dimensional mechanical imaging.

Some considerations must be addressed on the data reported. The frequency shift *ν*_B_ of the Brillouin scattered light is proportional to the material refractive index *n* and the local acoustic velocity V, which in turn is related to the mass density ρ and the real part of the longitudinal elastic modulus M′ (see Methods). Both density and refractive index are likely heterogeneous across the cells and variations in these two parameters may lead to a change in *ν*_B_. A previous analysis^45^ carried on the refractive index of HeLa cells reported *n* = 1.355–1.365 for the cellular nucleus, *n* = 1.36–1.39 for the cytoplasm and *n* = 1.375–1.385 for the nucleoli. These values reflect an overall mean variation in the refractive index (and thus in the Brillouin shift) of ∼1.5% amongst the different subcellular compartments of a HeLa cell. Although, the instrumental sensitivity would be capable of detecting those variations, the Brillouin frequency range measured in our experiments reflect higher changes more likely associated with variations in the longitudinal modulus M′. Moreover, it is also worth noticing that while the cellular nucleus has a lower refractive index compared to the cytoplasm, our Brillouin measurements show a higher nuclear frequency shift, suggesting that the refractive index heterogeneity in HeLa cells has a minor effect on the data reported. Similar changes in the refractive index are shown in another study^46^ that measured a mean value and a standard deviation of *n* = 1.376 ± 0.0035 in living HeLa cells, and *n* = 1.376 ± 0.0026 in 4% PFA fixed HeLa cells, reflecting a mean variation of 0.5% and 0.4%, respectively. To the best of our knowledge, there is a lack of information on the refractive index of stress granules, and so for the present study we have assumed that their mean value and variation are within the typical range reported for standard HeLa cells. This assumption can be further supported by the fact that the DIC images associated with the optical path difference do not show a relevant contrast between the stress granules and the surrounding cellular environment.

Besides the refractive index, an absolute value of the longitudinal modulus M′ also requires the exact knowledge of the local mass density of the medium. Although some studies estimated a mean cellular density of *ρ* = 1.080 g/mL (close to that of water)^47^, measurements of the local spatial variations of density across the different subcellular compartments and with characteristics comparable to those of our study have not been performed yet. As a result, the exact knowledge of the absolute value of M′ is a rather difficult task. Nonetheless, according to the findings reported in a previous study^22^, variations in the ratio *ρ*/*n*^2^ are substantially negligible across the cells. Although more detailed analyses will need to be performed to give an absolute value of M′, the above considerations motivate our assumption that variations in the Brillouin frequency shift can be directly associated with those in M′, which is a measure of stiffness at a high (GHz) frequencies.

In the present study, measurements on stress granules were performed using fixed cells. Previous AFM studies have found an increased stiffness in fixed cells compared to living cells^48–50^. While we found a similar trend after normalization to the cellular volume variations, our measurements showed that individual subcellular compartments have a similar frequency shift variation from living to 4% PFA fixed HeLa cells. These findings together with the measurements performed on the same cellular counterparts under appropriate control conditions should provide a sufficient basis to support our main hypothesis. Although, the BDB microscope is capable of acquiring mechanical images of living cells, the still relatively long (∼15 min) image acquisition time (see Methods) does not allow an accurate 3D localization of the stress granules, which are highly dynamic compartments that rapidly change both shape and location within the cellular volume^51^. In the future, we aim to repeat the experiments further investigating the effect of other proteins in living cells. However, this will require a substantial instrumental improvement in terms of the acquisition speed.

In conclusion, we described a method to efficiently enhance the contrast of single-stage Brillouin spectrometers using a simple diffraction mask. Imaging whole cells, we found altered biomechanics of stress granules in response to inclusion of mutant FUS protein. Our results suggest that the increased stiffness and viscosity of stress granules might lead to an aberrant stabilization, providing important insights about the onset of a critical liquid-to-solid phase transition previously proposed as a pathological trigger of the ALS disease. As such, our method paves the way for systematic and deep studies of the liquid-to-solid (glass) transitions in protein aggregates that are ubiquitous in neurodegenerative diseases.

## Methods

### Inelastic Brillouin scattering

Brillouin scattering is an inelastic scattering process arising in light interaction with local spontaneous acoustic waves of materials. The gain or loss of energy involved in this process results in a small shift in frequency of the scattered light, which in our backscattering geometry is given by *ν*_B_ = 4*nπV*/*λ*, where *λ* is the incident wavelength, *n* is the material refractive index and *V* = (M′/*ρ*)^0.5^ is the acoustic velocity, being *ρ* and M′ the density and the real part of the high-frequency longitudinal bulk modulus^28^. We should emphasize that while the Young’s modulus (E) obtained by standard elastography techniques is measured in a quasi-static regime, the longitudinal modulus is related to purely longitudinal waves of materials at GHz acoustic frequencies. Despite the different nature of these two moduli, a high correlation between variations of M′ and E has been consistently found under the same environmental conditions^22^, validating Brillouin light scattering as a reliable means to measure material stiffness.

### Confocal Brillouin microscope

The Brillouin microscope combines a scanning confocal imaging system with the background-deflection VIPA spectrometer (Supplementary Fig. 3). A CW single-longitudinal mode laser (Coherent Verdi, *λ* = 532 nm) was employed as the light source for all experiments. An oil-immersion objective lens (Olympus UPlanSApo ×100) of adjusted NA = 1 was used to focus and collect light in a backscattering geometry, providing a theoretical spatial resolution of ∼0.3 × 0.3 × 1.1 μm^3^. All samples were mounted on a motorized stage (Prior HLD117IX) to perform a rapid 3D sample scanning. The scattered light was collected by a single-mode optical fiber that worked as a confocal pinhole and delivered light to the spectrometer.

### Background-deflection VIPA spectrometer

The VIPA is a modified solid Fabry-Perot etalon that provides high (>50%) throughput efficiency through an anti-reflection coated entrance window that minimizes entrance losses^26^. In Brillouin microscopy, two or more crossed VIPA etalons are typically mounted in tandem to reach a contrast of ∼60 dB^39^, but multistage architectures suffer from a reduced throughput efficiency and system stability. In turn, our spectrometer integrates a single VIPA etalon (LightMachinery, OP-6721-3371-2) of R1 = 99.9% and R2 = 96% surface reflectivities and a custom diffraction mask with a rhomboidal aperture of 4 mm height of and 8 mm width at the output of the VIPA. Given the faster radial intensity decay of the diffraction pattern generated by a rhomboidal aperture with respect to the standard Airy diffraction pattern generated by circular apertures (Supplementary Fig. 1), which decays as J_1_(x)/x where J_1_ is a Bessel function of the first order, convolution of the VIPA transfer function with the mask diffraction pattern results in a 40 dB contrast enhancement.

### Cell culture and preparation

The cell line HeLa RFP-FUSP525L has been generated and maintained as described in supplemental material. For sample preparation, 50,000 cells were seeded in each μ-Dish 35 mm high Grid-50 Glass Bottom (ibidi) in 2 mL of culture medium. Where specified, 200 ng/mL doxycycline (Sigma-Aldrich) were added to the medium for RFP-FUSP525L induction. The next day, cells were treated with 0.5 mM sodium arsenite (Sigma-Aldrich) for 90 min, or left untreated, and then prepared as follows: washed with PBS (Sigma-Aldrich), fixed with 4% paraformaldehyde for 15 min at room temperature, washed with PBS, incubated 5 min with PBS containing 0.1 M glycine, washed two times with PBS and left in PBS for Brillouin acquisition.

### Cell immunofluorescence

To perform immunostaining after Brillouin acquisition, cells were permeabilized with PBS containing 0.1% Triton X-100 for 5 min, washed twice with PBS, incubate at least 30 min at room temperature with blocking solution (PBS/3%BSA/0.05% Tween20) and then overnight at 4 °C with primary antibody anti-PABP (1:200, SC-32318 Santa Cruz). The secondary antibody was a donkey anti-mouse Alexa Fluor 488 (1:200, Immunological Science). DAPI (Sigma-Aldrich) was used to stain nuclei. After final washes PBS was replaced with ibidi mounting medium.

### Validation measurements

Validation measurements were performed acquiring the Brillouin spectrum of an intralipid solution (Sigma-Aldrich, I141-100ML) at concentrations of 10%, simulating the experimental conditions found in materials of high turbidity.

### Cell imaging

Samples were imaged in DIC modality to select the cells of interest, with coordinates noted using engravings on the bottom of the dish. For live/fixed Brillouin measurements, live cells were left in their standard medium plus 10 mM HEPES (Gibco) and kept at 37 °C degrees inside a stage incubator (OkO Lab). After acquisition cells were fixed as described before and left in PBS. The same cells were then measured again. Brillouin images were acquired scanning the cells in the three-dimensions using the motorized stage with a transverse step size of 0.4 μm and axial step size of 1 μm. A Brillouin spectrum was acquired by a CCD camera (Photometrics Prime) at each scanning position with a data acquisition time of 100 ms and <10 mW optical power at the sample plane, resulting in a total image acquisition time of ∼15 min for a 2D cell plane. A spectral reference of distilled water was acquired before and after each measurement. The Brillouin maps were reconstructed fitting the acquired spectra with Lorentzian functions and evaluating the relative shift from water, which in the backscattering geometry has a well-known Brillouin shift of *ν*_B_ = 7.4 GHz^22^. Upon completion of the Brillouin acquisition, immunofluorescence was performed and dishes were transferred to an inverted confocal fluorescence microscope (Olympus IX83) equipped with an UPLSAPO ×60 oil immersion objective (NA = 1.35). The cells acquired in the Brillouin microscope were easily retrieved using the reference coordinates of the marked grid at the bottom of the ibidi dish. All fluorescence images were acquired through a confocal aperture of 110 μm with a ×2 zoom for a corresponding theoretical resolution of 0.1 × 0.1 × 0.7 μm^3^. The collinear light beams from a 405, 473, and 559 nm laser diode light source were injected into the microscope via a FV1200 MPE laser scanning confocal device. For the detection, standard setting for DAPI, Alexa Fluor 488 and RFP were used. An optical condenser (NA = 0.55) collected the transmitted light to the bright-field image detector for the DIC images. The 1024 × 1024 pixel fluorescence images (105.4 × 105.4 μm field of view) were collected in line sequential mode. Z-stacks were collected at 300 nm slice interval, for a total z depth of about 13–15 μm. The Olympus FV10 v.0402 software was used for confocal images analysis.

### Code availability

Custom codes based on MathWorks Matlab 2015 software were used for data acquisition and analysis. They can be accessed at https://github.com/manfredo89/Brillouin_2.

## Electronic supplementary material

Supplementary Information

Description of Additional Supplementary Items

Supplementary Movie 1

Supplementary Movie 2

## Data Availability

The datasets generated during and/or analyzed during the current study are available from the corresponding author on reasonable request.
