# Supplementary material for: Background-deflection Brillouin microscopy reveals altered biomechanics of intracellular stress granules by ALS protein FUS
Source: Commun Biol. 2018 Sep 10;1:139. doi: 10.1038/s42003-018-0148-x (PMC6131551; doi:10.1038/s42003-018-0148-x)
Supplement: Supplementary file 1 — Supplementary Information [file 42003_2018_148_MOESM1_ESM.pdf]

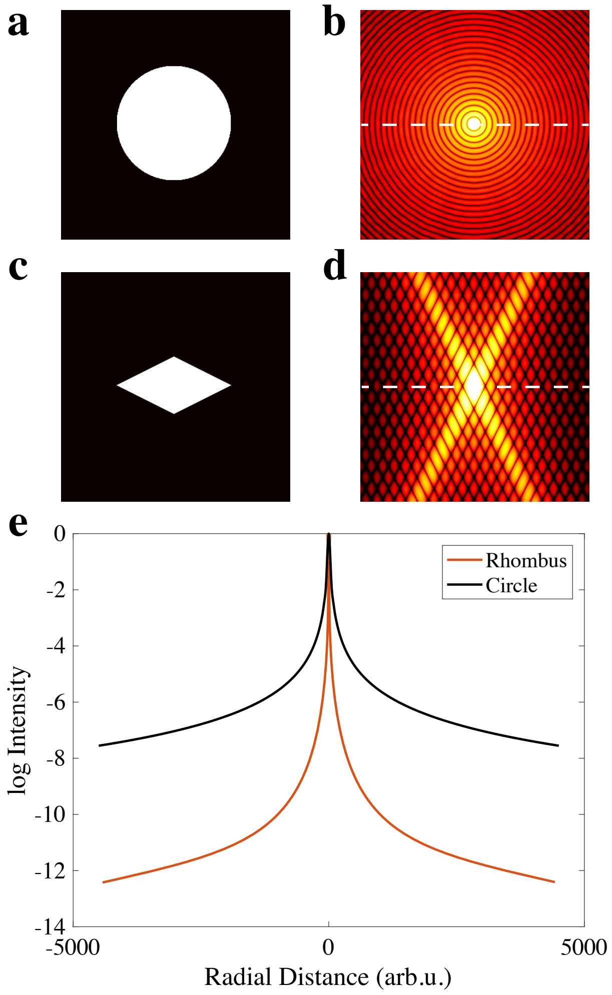

Supplementary Figure 1 – Fraunhofer diffraction for circular and rhomboidal apertures. Fraunhofer diffraction for a circular aperture (a) yields the well-known Airy disk pattern (b) characterized by a central peak and several concentric rings of slowly decaying intensity. On the other hand, a rhomboidal aperture (c) results in a radially asymmetric diffraction pattern with high-energy tails spreading along a perpendicular direction with respect to the rhombus sides (d). Logarithmic intensity profile along the direction (white dashed line) intersecting the rhombus edges (e). The diffraction pattern of the rhomboidal aperture manifests a faster intensity decay (red line) than the one given by the circular aperture (black line). Applied to the VIPA etalon configuration, the faster intensity decay given by the rhomboidal aperture gives a qualitative explanation of the enhanced spectral contrast.

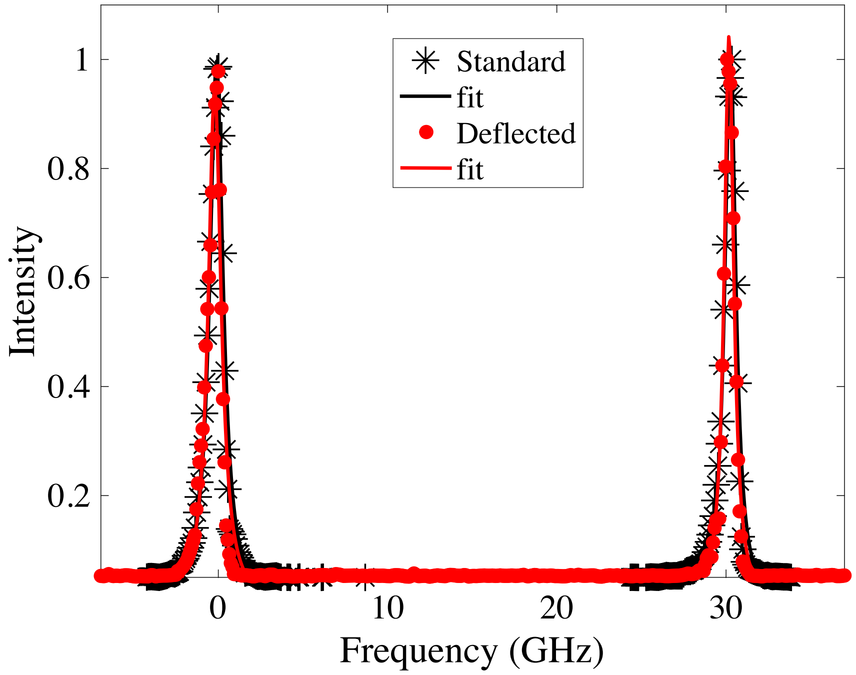

Supplementary Figure 2 – Spectral resolution. Normalized spectral intensity profiles for two consecutive interfering orders of the standard (black dots) and background-deflection (red dots) VIPA spectrometers along one FSR (30GHz). Peak shape and spectral resolution were not affected by the addition of the diffraction mask. By fitting the spectral profiles with a Lorenzian function (solid lines), we measured a spectral resolution of $\Delta\nu_s$=(675±12) MHz for the standard case, and $\Delta\nu_d$=(669±15) MHz for the background-deflection VIPA spectrometer.

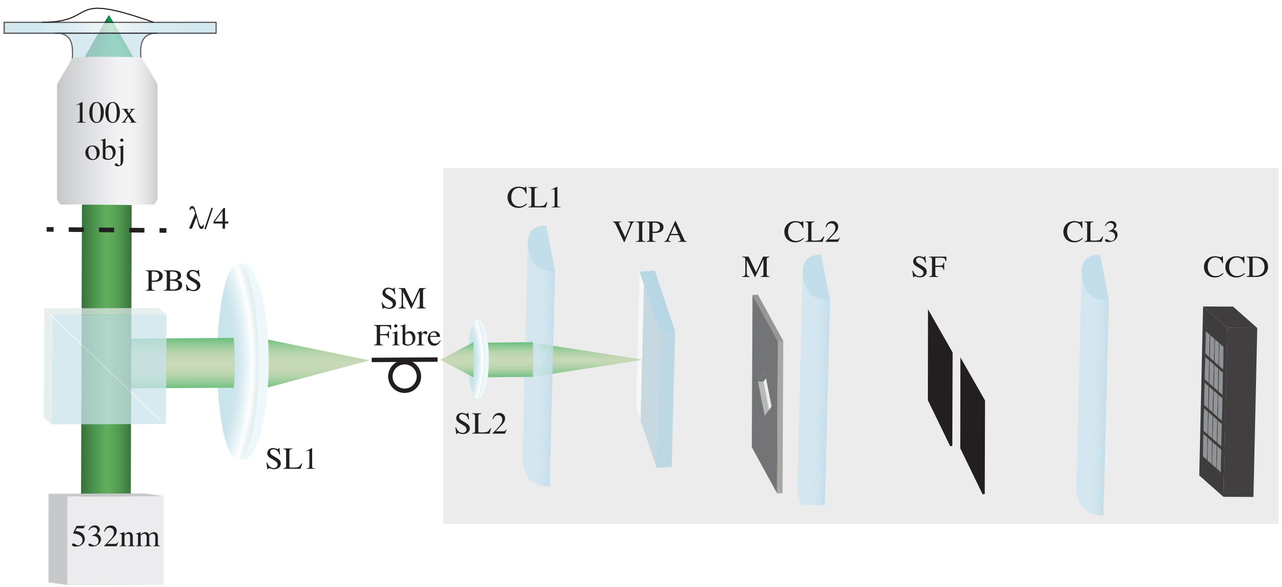

Supplementary Figure 3 – Schematic of the background-deflection Brillouin microscope. An expanded collimated beam from a single-longitudinal mode laser (Coherent Verdi V12) is focused by an objective lens to the sample. A polarizing beam splitter (PBS, Thorlabs PBSW-532) and a quarter wave plate (λ/4) were used to maximize the microscope illumination and collection efficiency. The backscattered light was coupled into a single-mode (SM) fiber (Throlabs P3-405B-FC-2) by a spherical lens (SL1). The SM fiber was used as a confocal pinhole and to deliver light to the spectrometer (grey envelope). At the fiber output, the beam was collimated by an aspheric lens (SL2, Thorlabs PAFA-X-4-A) and focused by a cylindrical lens (CL1, Throlabs LJ1653L1-A) of f=200mm focal length to the AR coated window of the VIPA. A second cylindrical lens (CL2, Throlabs LJ1653L1-A) of equal focal length performs a Fourier transform of the output field of the VIPA etalon. The resulting VIPA transfer (Airy) function is convoluted with the diffraction pattern of the mask (M) at the focal plane of CL2, where a spatial filter removes the elastic peaks to avoid camera saturation. The resulting spectral pattern is imaged by another cylindrical lens (CL3, Throlabs LJ1653L1-A) onto a CCD camera (Photometrics Prime).

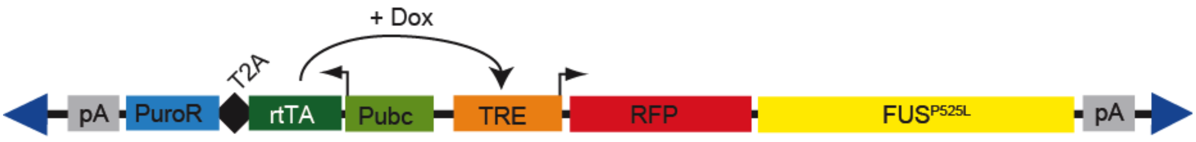

Supplementary Figure 4 – Schematic representation of the epB-Puro-TT-RFP-FUS^{P525L} construct. pA: polyadenylation signal; PuroR: puromycin resistance gene; T2A: self-cleavage peptide; rtTA: TET transactivator protein gene; Pubc: human Ubiquitin C constitutive promoter; TRE: TET responsive element; Dox: doxycycline; RFP: TagRFP. Blue triangles represent terminal repeats of the transposon.

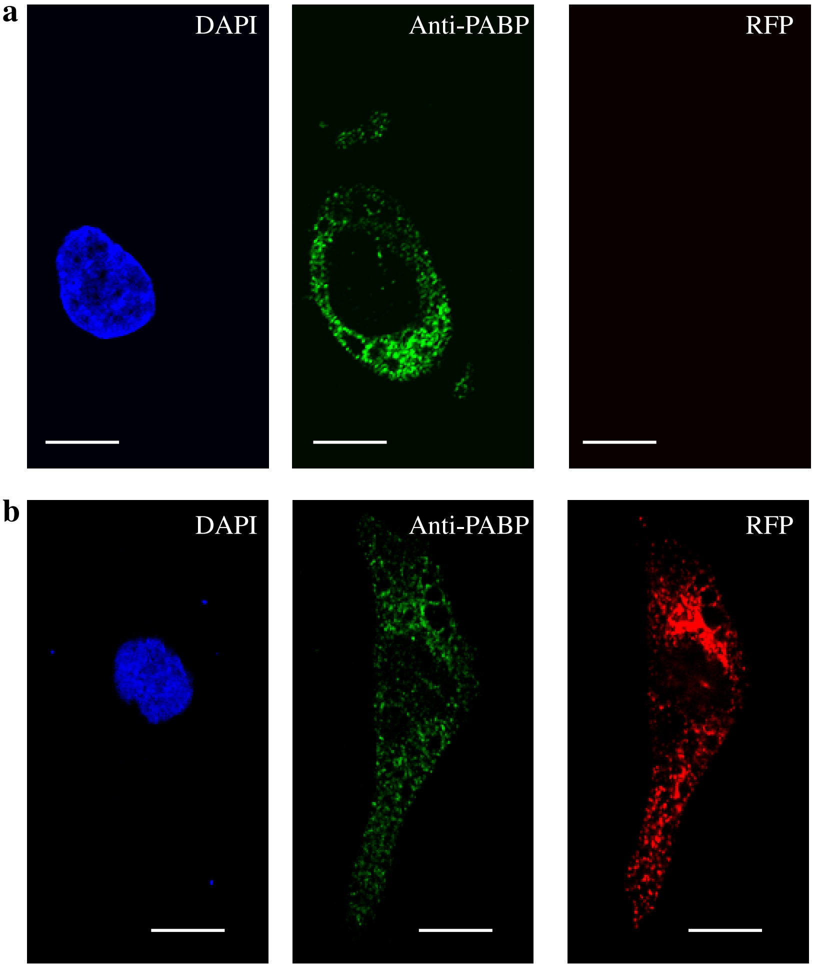

Supplementary Figure 5 – Confocal fluorescence images, related to Fig. 2, of HeLa cells untreated (a) or treated with doxycycline (b). DAPI (blue) labels the cell nucleus. Anti-PABP (green) recognizes this RNA-binding protein showing a diffused distribution in absence of cellular stress. The RFP panel shows the diffuse localization of RFP-FUS^{P525L}, present only in doxycycline-induced cells. Scale bars are 10µm.

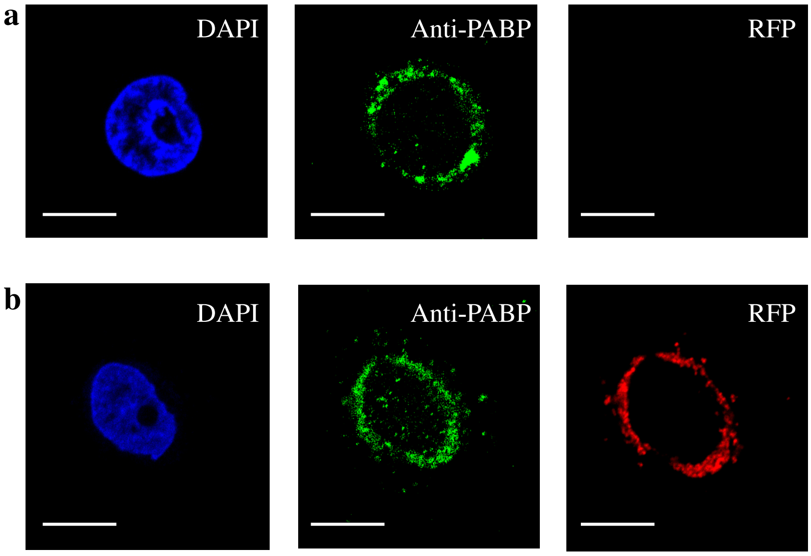

Supplementary Figure 6 – Confocal fluorescence images, related to Fig. 3, of HeLa cells untreated (a) or treated with doxycycline (b), and exposed to sodium arsenite to induce oxidative stress. DAPI (blue) labels the cell nucleus. Anti-PABP (green) recognizes this RNA-binding protein marking SGs under stress conditions. The RFP panel shows the recruitment of RFP-FUS^{P525L}, present only in doxycycline-induced cells (b), into SGs. Scale bars are 10µm.

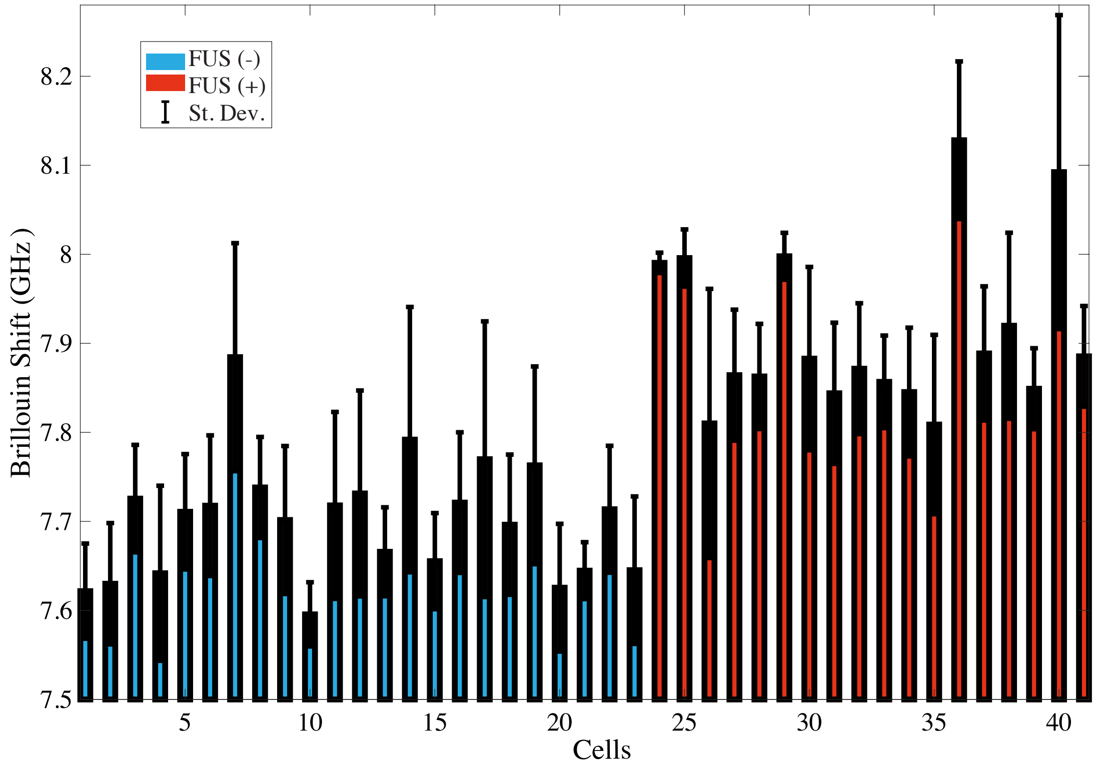

Supplementary Figure 7 – Bar plot of the mean Brillouin shift measured into SGs for each cell with (red) and without (green) RFP-FUS^{P525L} expression. Error bars are standard deviations.

Supplementary Note 1 - Generation and maintenance of the HeLa RFP-FUS^{P525L} cell line

HeLa cells were maintained in DMEM-F12 supplemented with 10% FBS, 1x Penicillin/Streptomycin (all from Sigma-Aldrich) and 1x Glutamax (Life Technologies). The epB-Puro-TT-RFP-FUS^{P525L} plasmid was generated by inserting the coding sequence of human FUS, containing the pathogenic P525L mutation [NM_004960.3(FUS):c.1574C>T (p.Pro525Leu)], in a modified version of the enhanced piggyBac transposable vector epB-Puro-TT, downstream and in frame with the TagRFP coding sequence [1] (Supplementary Fig. S4) The resulting construct contains the piggyBac terminal repeats flanking a constitutive cassette driving the expression of the Puromycin resistance gene fused to the rtTA gene and, in the opposite direction, a tetracycline-responsive promoter element (TRE) driving the conditional expression of the transgene. HeLa cells were co-transfected with epB-Puro-TT-RFP-FUS^{P525L} and the piggyBac transposase as described [2], using the Lipofectamine 2000 (Life Technologies) following manufacturer's instructions. Selection in 1 μg/ml puromycin gave rise to a stable and inducible cell line.

Supplementary references

[1] Rosa, A., Papaioannou, M.D., Krzyspiak, J.E., Brivanlou, A.H., miR-373 is regulated by TGFβ signaling and promotes mesendoderm differentiation in human Embryonic Stem Cells. Dev Biol. **391**(1), 81–88 (2014).

[2] J. Lenzi, et.al. Differentiation of control and ALS mutant human iPSCs into functional skeletal muscle cells, a tool for the study of neuromuscolar diseases. Stem Cell Research **17**(1), 140-7 (2016).
