# Supplementary material for: Background-deflection Brillouin microscopy reveals altered biomechanics of intracellular stress granules by ALS protein FUS
Source: Commun Biol. 2018 Sep 10;1:139. doi: 10.1038/s42003-018-0148-x (PMC6131551; doi:10.1038/s42003-018-0148-x)
Supplement: Supplementary file 2 — Description of Additional Supplementary Items [file 42003_2018_148_MOESM2_ESM.pdf]

Description of additional supplementary items

Supplementary Video 1

3D imaging in Brillouin, fluorescence and DIC modalities (from left to right) of a single stressed HeLa cell without mutant FUS expression. Z-stacks were taken at 1 um depth from each other.

Supplementary Video 2

3D imaging in Brillouin, fluorescence and DIC modalities (from left to right) of a single stressed HeLa cell with mutant FUS expression. Z-stacks were taken at 1 um depth from each other.
